# Identification, Characterization and Functional Analysis of Fibroblast Growth Factors in Black Rockfish (*Sebastes schlegelii*)

**DOI:** 10.3390/ijms24043626

**Published:** 2023-02-11

**Authors:** Chaofan Jin, Kai Yan, Mengya Wang, Weihao Song, Xiangfu Kong, Zhengrui Zhang

**Affiliations:** 1 MOE Key Laboratory of Marine Genetics and Breeding, College of Marine Life Sciences, Ocean University of China, Qingdao 266003, China; 2 Laboratory of Tropical Marine Germplasm Resources and Breeding Engineering, Sanya Oceanographic Institution, Ocean University of China, Sanya 572000, China

**Keywords:** fibroblast growth factors, muscle development, gonad development, *FGF1*, *Sebastes schlegelii*

## Abstract

Fibroblast growth factors (FGFs) are short polypeptides that play essential roles in various cellular biological processes, including cell migration, proliferation, and differentiation, as well as tissue regeneration, immune response, and organogenesis. However, studies focusing on the characterization and function of FGF genes in teleost fishes are still limited. In this study, we identified and characterized expression patterns of 24 FGF genes in various tissues of embryonic and adult specimens of the black rockfish (*Sebates schlegelii*). Nine FGF genes were found to play essential roles in myoblast differentiation, as well as muscle development and recovery in juvelines of *S. schlegelii*. Moreover, sex-biased expression pattern of multiple FGF genes was recorded in the species’ gonads during its development. Among them, expression of the *FGF1* gene was recorded in interstitial and sertoli cells of testes, promoting germ-cell proliferation and differentiation. In sum, the obtained results enabled systematic and functional characterization of FGF genes in *S. schlegelii*, laying a foundation for further studies on FGF genes in other large teleost fishes.

## 1. Introduction

Fibroblast growth factors (FGFs) are short peptide growth factors that contain a core region composed with six highly conserved (30–60% homology) subunits of 120 amino acid residues among different FGF genes [1,2,3]. Due to high affinity to heparin, FGFs interact with cell-surface heparan sulfate proteases, playing vital roles in signal transduction [4]. In the 1970s, *FGF1* and *FGF2* were identified to be the first two members of the FGF gene family [5].To date, FGF family genes have been identified in numerous vertebrates and invertebrates. Two of them (*EGL-17* and *LET-756*) have been identified in *Caenorhabditis elegans*, which is the most primitive postnatal animal that possesses FGF genes [6]. Moreover, six and seven FGF genes have been identified and isolated from *Ciona intestinali* and *Branchiostma florida*, respectively. In vertebrates, the FGF genes have been mainly identified and isolated from human (22 genes) and zebrafish (27 genes) genomes [3,7]. In addition to characters of high affinity for heparin and interaction with cell-surface heparan sulfate proteases, FGF genes also contain a core region consisting of six subunits of 120 amino acid residues, which is conserved among different FGF genes, with 30%-60% homology [8].

FGF family members have proven to be crucial for multiple biological processes, including cell migration, proliferation and differentiation, tissue regeneration, immune response, and organogenesis [9]. In mammals, *FGF1* and *FGF2* have been identified to be crucial for neural cell migration [10]. Moreover, *FGF4* and *FGF7* could promote the migration of myoblasts and keratinocytes, respectively [11,12]. For cell differentiation, multiple FGF genes are associated with stem-cell differentiation [13]. *FGF2* is presumed to promote cell proliferation in multiple tissues, including brain, muscles, and gonads. Furthermore, the exogenous *FGF2* protein has been widely utilized for cell cultures in vitro to promote cell proliferation [14,15,16]. The involvement of the FGF genes in other biological processes of FGF genes has been demonstrated in multiple species, including human, mouse and rabbit [17,18,19]. In fishes, FGFs have also been widely identified to be involved in multiple biological processes. For example, Fgf signaling has been shown to participate in electrosensory versus mechanosensory lateral line organ development in non-teleost ray-finned fish [20]. In stickleback, the expression profiles of *FGF8a* and *FGF8b* during embryo development suggest their functional divergence. Moreover, *FGF16* and *FGF24* function in teleost pectoral appendage [21].

In the aquaculture industry, the growth rate of farmed fish is commonly dependent on skeletal muscle growth. In recent years, more and more studies investigate the molecular mechanisms of muscle development, and a series of genes have been associated with muscle growth in fish [22,23]. For example, FGF has been identified to regulate dedifferentiation during skeletal muscle regeneration in adult zebrafish [24]. Furthermore, FGF-driven Tbx protein activities directly induce myf5 and myod to initiate zebrafish myogenesis [25]. However, there are few studies focused on the relationship between FGF genes and teleost muscle growth in non-model species. Proper gonad development is also important, as it determines the quality of gametes of produced fishes in aquaculture. The gametes are vital germplasm resources and genetic information transmitters of high-quality breeds [26,27]. Moreover, a recent study has demonstrated a shift of nutrients and energy from body growth to gonadal development during sexual maturation in salmonids [28]. Although *FGF* genes have been widely identified to play vital roles in gonad development in mammals and zebrafish [29,30], our knowledge of the expression and functions of FGF genes in gonad development is limited in non-model teleost fishes.

Black rockfish (*Sebastes schlegeli*) is an important marine teleost fish species with huge economic importance in the western North Pacific. The postnatal growth of *S. schlegelii* exhibits an obvious indeterminate growth pattern, including hyperplasia and hypertrophy of muscle fibers, with sexual dimorphism (unpublished data). High-quality chromosome-level genome sequencing provides a useful tool to perform comprehensive gene analysis [31]. In our previous studies, we have demonstrated that *pax3*/*pax7* could be reliable markers for muscle satellite cells and function in the repair of muscle, as well as myosin genes, participating in muscle development via promoting myoblast proliferation or differentiation [32,33]. Here, we identified a total of 24 FGF genes in *S. schlegelii* and analyzed their dynamic expressions in various tissues of *S. schledelli* at different developmental stages. To determine the role and involvement of FGF genes in muscle development, myoblast differentiation, and muscle regeneration, transcriptome data and qRT-PCR analyses were performed. Our study indicated that FGF genes were differentially regulated during gonad development and identified several FGF genes with sex-biased expression patterns in gonads. *FGF1* was mainly expressed in intestinal cells and sertoli cells in testis. Our results indicate that the *S. schlegelii* FGF1 protein could promote the expression levels of genes related to germ-cell proliferation and differentiation. Taken together, our results lay the foundation and provide valuable insights for further functional analysis of FGF genes in teleost species.

## 2. Results

### 2.1. Identification of FGF Genes in S. schlegelii

In total, 24 FGF family genes were identified from the *S. schlegelii* genome and transcriptome database. The amino acid sequences of FGF proteins ranged from 139 to 286 amino acids, and the molecular weight of FGF proteins ranged from 16.36 kDa to 32.20 kDa, with the isoelectric points ranging from 5.67 to 10.76. Detailed information of the FGF genes is provided in Table 1. 

**Figure 1:**
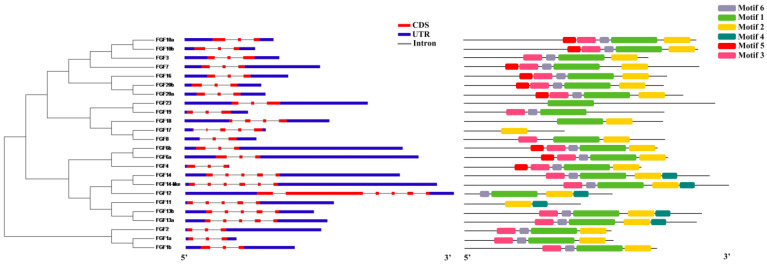
Gene structure and motif analysis of FGF genes. The phylogenetic analysis of FGF genes was based on the maximum likelihood method (**left panel**). The exon–intron strictures of FGF genes (**the middle panel**), and the red and blue boxes represented exons and UTRs, respectively. Motif analysis of FGF genes (**right panel**). The boxes with different colors represented different motifs.

Functional domain analysis showed that all *S. schlegelii* FGF genes had an N-terminal FGF domain (Figure S1). To characterize the structures of FGF genes, we aligned the transcriptome with genome sequences of FGF genes following GT-AG rule. The result showed FGF genes with three to six exons (Figure 1, left). Motif analysis showed that most FGF genes had motif 1, except *FGF11*, *FGF 17*, and *FGF23*.

**Figure 2:**
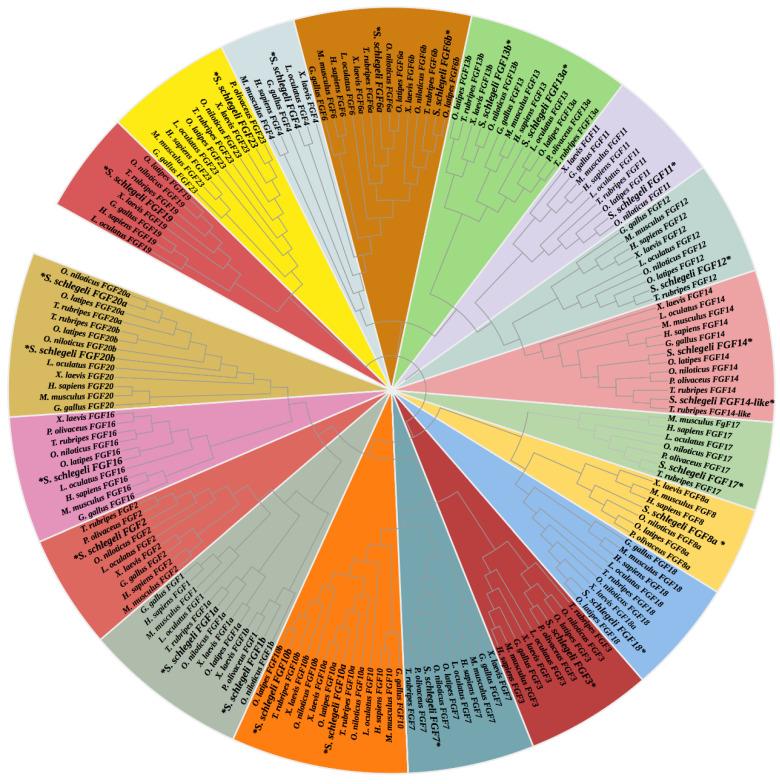
The phylogenetic analysis of FGF genes. The Neighbor-Joining method (bootstrap = 1000) was utilized for the construction of phylogenetic tree by MEGA 7.0 software.

To explore the phylogenetic relationships of FGF genes, the phylogenetic analysis of FGF genes was conducted. As shown in Figure 2, 24 *S. schlegelii* FGF genes were classified into 18 subfamily clusters with six duplicated genes: *FGF1* (*FGF1a* and *FGF1b*), *FGF6* (*FGF6a* and *FGF6b*), *FGF10* (*FGF10a* and *FGF10b*), *FGF13* (*FGF13a* and *FGF13b*), *FGF14* (*FGF14a* and *FGF14b*), and *FGF20* (*FGF20a* and *FGF20b*). Synteny analysis demonstrated that the subtypes of above duplicated genes were generated by teleost genome duplication, which showed high conservation among teleost (Figure S2).

### 2.2. Expression Patterns of FGF Genes in Adult Tissues and Embryos

**Figure 3:**
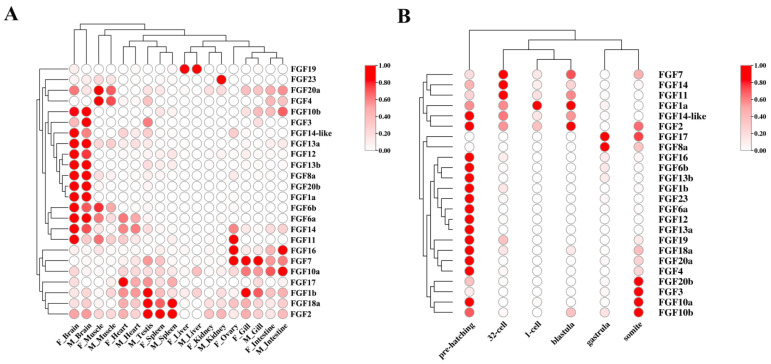
Expression patterns of FGF genes in adult tissues (**A**) and embryo developmental stages (**B**). The color scale from white to red indicates the TPM value from low to high.

The expression levels of *S. schlegelii* FGF genes in adult tissues and embryos at different developmental stages were determined based on the TPM values from 89 transcriptomes (CNGB Nucleotide Sequence Archive, accession ID: CNP0000222). In adult tissues (Figure 3A), FGF genes showed divergent expression patterns. For example, multiple FGFs, such as *FGF10b*, *FGF3*, *FGF14-like*, *FGF13a*, *FGF13b*, *FGF12*, *FGF8a*, *FGF20b*, and *FGF1a*, showed extremely high expression levels in both male and female brains, while *FGF19* and *FGF23* were highly expressed in the liver and kidney, respectively. Moreover, the remaining FGF genes showed relatively generic expression patterns in various tissues. 

During the early developmental stages of *S. chlegelii*, FGF genes showed dynamic expression patterns. As shown in Figure 3B, most FGF genes were significantly up-regulated at pre-hatching stage. Furthermore, the expression levels of several FGF genes were extremely high at the somite stage, including *FGF20b*, *FGF3*, *FGF10a*, and *FGF10b*, whereas *FGF17* and *FGF8a* were preferentially expressed at the gastrula stage. Moreover, some FGF genes showed relatively high expression levels until the blastula stage, then decreased at the gastrula stage and increased at the somite stage or pre-hatching stage. 

### 2.3. S. schlegelii FGF Genes Were Involved in Muscle Cell Differentiation In Vitro, Muscle Development of Juvenile Fish, and Injured Muscle Repair

**Figure 4:**
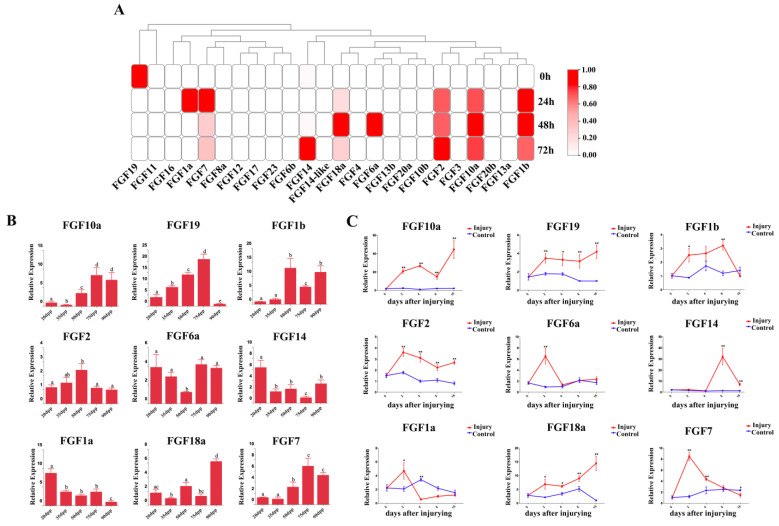
The involvement of *S. schlegelii* FGF genes in myoblast differentiation, muscle development, and muscle injury repair. (**A**) The expression alterations of FGF genes during the differentiation process of muscle cells in vitro. The TPM value is indicated by color scale. The color from white to red represents the TPM value from low to high. (**B**) The expression profiles of 9 FGF genes during muscle development in juvenile fish. Different letters indicated statistical significance (*p* < 0.05). (**C**) The relative expression levels of 9 FGF genes at different time-points after injury. Vertical bars represent the mean ± SEM (n = 3). * indicates *p* < 0.05, and ** represents *p* < 0.01. All data of qRT-PCR are shown as mean ± SEM (n = 3).

FGF genes have been widely identified to be crucial for muscle development. In this study, we determined the transcriptional signature of FGF genes during muscle-cell differentiation in vitro based on the transcriptome database built in our previous study to investigate the potential roles of FGF genes during muscle-cell differentiation. As shown in Figure 4A, nine FGF genes were found to be differentially regulated during this process. Among them, *FGF19* was down-regulated from the beginning of muscle-cell differentiation, while *FGF2*, *FGF10a* and *FGF1b* were significantly up-regulated at 24 h after differentiation induction. Furthermore, *FGF1a* and *FGF7* showed the highest expression levels at 24 h post-induction of differentiation, whereas the expressions of *FGF18a* and *FGF6a* reached their highest points at 48 h post-induction of differentiation. Moreover, the expression of *FGF14* could be detected until 72 h post-induction of differentiation. 

Thus, the involvement in muscle-cell development in vitro of the above nine FGF genes has been identified; however, their function in muscle development in vivo remains unknown. Here, the expression patterns of these nine FGF genes during juvenile muscle development (20dpp (days post parturition), 35dpp, 50dpp, 75dpp, and 90dpp) were characterized by qRT-PCR (Figure 4B). *FGF10a*, *FGF1b*, *FGF18a*, and *FGF7* appeared to be up-regulated from 20dpp to 90 dpp, while *FGF14* and *FGF1a* were down-regulated during this process. Furthermore, *FGF19* was up-regulated and had the highest expression level at 75 dpp and then was down-regulated at 90 dpp. The expression of *FGF2* reached the highest point at 50 dpp and then decreased, whereas *FGF6a* showed the lowest expression level at 50 dpp.

Muscle injury repair is an ideal model that is commonly utilized to investigate the rule of muscle-cell proliferation, differentiation, and the function of specific genes in muscle regeneration. In this study, the expression dynamics of the above nine FGF genes during the process of injured muscle repair were investigated by qRT-PCR. The results showed that the expressions of selected FGF genes were dynamically regulated during the muscle regeneration process after injury (Figure 4C). Compared to the control group, most FGF genes, including *FGF10a*, *FGF10a*, *FGF2*, and *FGF18a*, showed relatively higher expression levels in the injury group at each time-point. The expression levels of *FGF6a*, *FGF1a*, and *FGF7* reached the highest points at 2 dpi (days post-injury) and then decreased. Moreover, *FGF1b* was significantly up-regulated during 0–8 dpi, while the expression of *FGF14* showed no significant change compared to the control group during 0–4 dpi, increased rapidly at 8 dpi, and then decreased at 16 dpi. In addition, protein–protein interaction analysis was performed using a string database. The results demonstrated that there were direct or indirect interactions between the above nine FGF genes and some muscle-development-related genes like *pax3*, *pax7*, *myh2*, *myod1*, *myo*,*g* and *myf5* (Figure S3).

### 2.4. Transcriptional Signature of S. schlegelii FGF Genes during Gonad Development 

**Figure 5:**
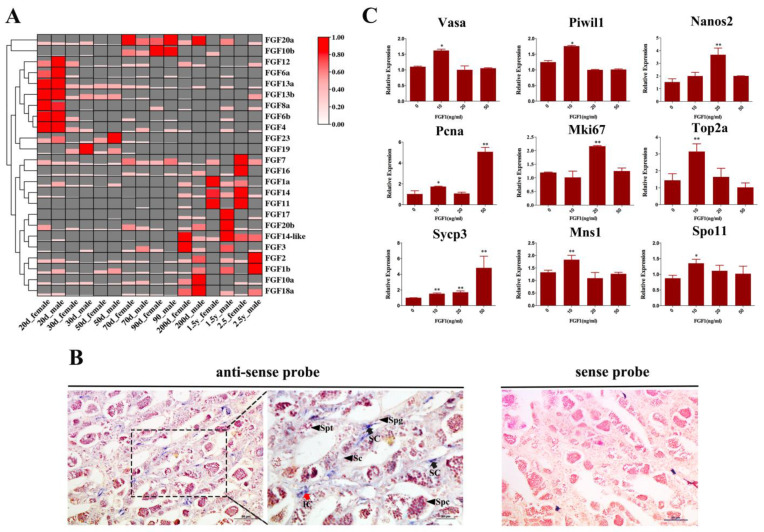
The involvement in gonad development of *S. schlegelii* FGF genes. (**A**) The expression patterns of FGF genes during gonad development during gonad development. The color scale and the height scale of columns represent the TPM value. The red color and high column represent the relatively higher TPM value. The white color and low column represent the relatively lower TPM value. (**B**) The expression patterns of *FGF1* transcripts in the testis. Positive signals were observed in interstitial cells (red arrows) and sertoli cells (black arrows). Sc, spermatogenetic cysts; Spg, spermatogonia; Spc, spermatocytes; Spt, spermatid; SC, sertoli cell; IC, intestinal cell. (**C**) The expression levels of germ-cell-related genes with FGF1 protein (10ng/mL, 20ng/mL and 50 ng/mL) treatment. Vertical bars represent the mean ± SEM (n = 3). *, ** on the top of column indicates statistical significance (*p* < 0.05, *p* < 0.01) between treatment group and control group.

Based on the comprehensive transcriptome databases of gonads at different stages, we explored the expression profiles of FGF genes during the development of both testis and ovary. The results showed that FGF genes exhibited disparate expression patterns in the gonads at different developmental stages (Figure 5A). For instance, *FGF12*, *FGF6a*, *FGF13a*, *FGF13b*, *FGF8a*, *FGF6b*, and *FGF4* were most abundant in both testis and ovary at 20 dpp, while *FGF20a* showed relatively high expression levels from 70 dpp to 200 dpp, Moreover, we found that multiple FGF genes started to be expressed from 200 dpp, and some showed obvious sex-biased expression patterns. For example, *FGF18a*, *FGF1b*, and *FGF2* were highly expressed in the testis compared to ovary, whereas *FGF7*, *FGF16*, *FGF1a*, *FGF14*, and *FGF1* exhibited ovary-biased expression patterns.

### 2.5. The Location and Function of FGF1 in S. schlegelii Testis

We have described the biased expression patterns of FGF gene in male and female gonads; nevertheless, their functions in gonads remain unknown. Here, we selected *FGF1*, which showed similar expression patterns as *FGF2*, the factor that is well-known to be crucial for male germ-cell development in multiple species, to investigate its potential roles in the testis. First, *FGF1* mRNA was identified to be mainly located in intestinal cells and sertoli cells of testis by ISH (Figure 5B). Then, the purified FGF1 protein (Figure S4) was utilized to incubate the testis in vitro to investigate its effects on male germ-cell development. As shown in Figure 5C, *nanos2*, *vasa*, and *piwil1*, the markers of early germ cells, were up-regulated with 10ng/mL FGF1 protein treatment; meanwhile, *pcna*, *top2a*, and *mki67* (the germ-cell proliferation marker), were also up-regulated with the incubation of FGF1 protein. Moreover, FGF1 protein could also promote the expressions of several spermatocyte makers, including *sycp3*, *spo11*, and *mns1*. The results above demonstrate that testis-biased *FGF1* genes, which are expressed in intestinal cells and sertoli cells, could have functions for male germ-cell proliferation and differentiation.

## 3. Discussion

### 3.1. The Dynamic Functions of FGF Genes in S. schlegelii

In this study, we identified 24 FGF genes and characterized their expression patterns in 10 adult tissues and six embryo developmental stages based on comprehensive transcriptome databases. The results showed that FGF genes were ubiquitously expressed in different tissues and developmental stages with divergent expressions, suggesting that FGFs are widely involved in various biological processes and perform multiple biological functions in *S. schlegelii*, as reported in mammals. For instance, *FGF19*, was found to be specifically expressed in the liver of *S. schlegelii*, implying that it may be involved in liver physiological function, including glycogen metabolism [34], lipid metabolism [35], and immune response [36], as demonstrated in mammals. In zebrafish, FGF signaling has also been widely verified to play a key role in liver homeostasis regulation and specification [37,38]. In addition, we found that multiple *S. schlegelii* FGF genes were abundant in brain, which was consistent with the fact that the FGF genes are closely related to the nervous system. For example, *FGF13* has been shown to be extremely abundant in brain and crucial for the nervous system in human [39]. In teleost, FGFs could promote the effects of IGF-1 on the proliferation of rod precursor cells [40]. Moreover, a previous study demonstrated that exogenous *FGF20* could promote neural stem-cell differentiation into dopamine neurons in monkeys [41]. Consistent with a previous study showing that *FGF7* is important for the function of the middle ear [42], which is the homologous organ to fish gills, *S. schlegelii FGF7* exhibited high expressions in gills. Taken together, the FGF genes may participate in various biological processes and conduct conserved functions between mammals and teleost. It should be noted that multiple FGF genes were highly expressed at the pre-hatching stage during embryo development [43], suggesting their potential roles in this process; of course, further exploration is needed to confirm this possibility. 

### 3.2. The Involvement of FGFs in Myoblast Differentiation, Muscle Development, and Muscle Regeneration

The proliferation and differentiation of myoblasts contribute to the development of skeletal muscles [44]. In the past decade, many strategies have been attempted to promote muscle growth, and molecules associated with muscle growth have been widely identified. It is widely known that FGF genes play a vital part in muscle development and growth via promoting cell proliferation and differentiation. For example, *FGF10* is required for smooth muscle cell progenitors differentiating into smooth muscle cell lineages [45]. *FGF6* has been identified to function in stimulating myoblast proliferation/migration and muscle differentiation/hypertrophy [46]. Moreover, it has been shown that zebrafish *FGF2* could promote vascular smooth muscle cell migration to platelet-derived growth factor [47]. Here, we summarized the expression profiles of FGF genes during myoblast differentiation in vitro. The current results show that nine FGF genes had divergent expressions at different times of myoblast differentiation (Figure 4A). Most selected FGF genes were up-regulated with myoblast differentiation, suggesting their potential roles in myoblast differentiation. Interestingly, *FGF2*, the more well-known growth factor, could inhibit myoblast cell differentiation and was significantly up-regulated during myoblast differentiation of *S. schlegelii*. It was reported that endogenous and exogenous *FGF-1* differentially regulate the myogenic differentiation of L6–10 cells [48]. Therefore, we speculated that myoblast proliferation and differentiation could be differentially regulated by endogenous and exogenous FGF genes of *S. schlegelii*. 

In teleost, the research focusing on FGF functions in muscle is most developed for zebrafish. FMedaka *FGF6* has also been identified to be involved in the continuous generation of muscle fibers within the myotomal musculature [49]. The relationship between FGF genes and muscle growth in non-model teleost has only been described in few species. In grass carp, *FGF6* was found to be positively correlated with fiber diameter and plays an important part in muscle-growth regulation [50]. Here, *S. schlegelii* FGF genes showing a response to myoblast differentiation were selected to investigate the potential functions of FGF genes in muscle growth in vivo. Our current results show that all selected FGF genes were dynamically expressed during the muscle development of juvenile *S.schlegelii* (from 20 dpp to 90 dpp) (Figure 4B), among which, the expression levels of *FGF10a*, *FGF19*, *FGF6a*, and *FGF7* were found to significantly increase from 50 dpp to 75 dpp, corresponding to the periods during which the number and size of muscle fibers significantly increased [33], indicating their vital roles in contributing to the muscle growth. 

Muscle injury repair is a perfect model to investigate the mechanisms of muscle regeneration and the functions of specific genes. We previously established the muscle injury repair model of *S. schlegelii* and described the expression patterns of *pax3* and *pax7*, the markers of satellite cells, during muscle regeneration [32]. FGF genes have been proven to be related to muscle regeneration in multiple species, such as human, mouse, and zebrafish [4,24,51]. In this study, we explored the expression patterns of selected *S. schlegelii* FGF genes at different time-points post-muscle-injury (Figure 4C). Our results showed that FGF genes were dynamically regulated during muscle regeneration, suggesting that FGF genes might work at different times during muscle regeneration. 

### 3.3. The Involvement of S. schlegelii FGF Genes in Gonad Development

FGF genes have been widely known to be crucial for gonad development in vertebrates. For instance, cell-intrinsic FGF signaling contributes to PGCs homing in and early somatic gonad development in zebrafish [30,52]. In an avian model, it was found that FGF, insulin, and SMAD signaling cooperate for PGC self-renewal [53]. In *S. schlegelii*, multiple FGF genes also exhibited high expression in the early gonad, indicating that they might participate in PGC homing, proliferation, differentiation, and early gonad development. Furthermore, several FGF genes showed obvious sex-biased expression patterns in gonads after the sex determination period (200 dpp, 1.5 year, and 2.5 year). In teleost, the sex-biased expression pattern of FGF genes in gonads was reported in a previous study showing that *FGF16* was abundant in ovary compared to testis during gonad development in tilapia [54]. Combining our results and previous studies, we speculated that FGF genes could be functional in gonad development with a sex bias. Among these genes, *FGF2* is well-characterized and has been shown to be essential for spermatogonia development and culture in vitro [16]. Here, we performed ISH analysis to investigate the location of *FGF1*, which showed similar expression patterns with *FGF2*, in *S. schlegelii* testis and explored its effects on testis development in vitro. The results show that *FGF1* was highly expressed in intestinal cells and the sertoli cells of testis, consistent with the results in roe deer [55]. The functions of FGF1 in the testis have been demonstrated in several mammal species. For instance, FGF1 is important for leydig cell development in rat [56]. In roe deer, FGF1 protein could function in the interaction between sertoli cell and spermatid [55]. In our study, the function analysis demonstrated that purified FGF1 protein of *S. schlegelii* promotes the expression levels of germ cell genes, including *vasa*, *piwil1*, *nanos2*, *pcna*, *mki67*, *top2a*, *spo11*, *sycp3* and *mns1*. *Vasa*, *nanos2*, and *piwil1* have been shown to the markers of early germ cells [57,58]. *Pcna*, *mik67*, and *top2a* are known as markers for cell proliferation [59,60]; meanwhile, *Spo11*, *sycp3*, and *mns1* are known to be expressed in meiotic cells [61]. Thus, we speculated that FGF1 protein secreted from somatic cells could stimulate the spermatogenesis by promoting germ-cell proliferation and differentiation.

## 4. Materials and Methods

### 4.1. Identification and Characterization of FGF Family Genes in S. schlegelii

To identify FGF family genes in *S. schlegelii*, the genome and transcriptome databases, which are available at CNSA (CNGB Nucleotide Sequence Archive) under the accession ID CNP0000222, were searched against the FGF amino acid sequences from some representative species, including *H. sapiens*, *M. musculus*, *O. latipes*, *O. niloticus*, *L. oculatus*, and *G. aculeatus*. A TBLASTN algorithm search was performed, and sequences with e-values below e-5 were collected. Then, the putative FGF sequences were used as queries in NCBI BLAST (https://blast.ncbi.nlm.nih.gov/Blast.cgi?CMD=Web&PAGE_TYPE=BlastHome (22 July 2022). Only the sequences that got hits in at least one transcriptome were regarded as candidate FGF genes; then, all *S. schlegelii* FGFs sequences were saved in fasta format. The FGF domain was predicted by SMART [62] and Pfam (http://pfam.xfam.org (25 July 2022)) based on *S. schlegelii* FGFs amino acid sequences in a fasta format. The potential cellular location of the FGF proteins of *S. schlegelii* was predicted by WoLF PSORT (https://www.genscript.com/psort/wolf_psort.html (25 July 2022)) using amino acid sequences. The coding strand and chromosome location of FGF genes were concluded by a genome annotation file. The ExPasy site (https://web.expasy.org/protparam/ (25 July 2022)) was utilized to calculate the molecular weight (Mw) and theoretical isoelectric point (pl) of FGF proteins in *S. schlegelii* using amino acid sequences. The motif analysis of FGF proteins was performed with the Motif Elicitation (MEME) program (http://meme-suite.org/tools/meme (25 July 2022)). The gene structure of FGF genes was determined by comparison of transcriptome and genome data and displayed using CSDS 2.0 program (http://gsds.cbi.pku.edu.cn (27 July 2022)). 

### 4.2. Phylogenetic and Synteny Analysis of FGFs in S. schlegelii 

The amino acid sequences of FGFs from nine species were downloaded from the NCBI (https://www.ncbi.nlm.nih.gov/ (22 July 2022)) and *Ensembl* (http://www.ensembl.org/index.html (22 July 2022)) databases. Then, the multiple amino acid sequences alignments of FGF proteins from nine species and *S. schlegelii* was conducted via Clustal W [63]. Then, the phylogenetic analysis of FGF proteins was performed using the Neighbor-Joining method by MEGA 7.0 [64] and visualized by the iTOL website (https://itol.embl.de (26 July 2022)). The synteny analysis of FGF genes on the chromosome among mammals and teleost was conducted using the genomics website (http://www.genomicus.biologie.ens.fr/genomicus (29 July 2022)). Then, the schematic diagram was drawn by hand. 

### 4.3. Transcriptome-Based Analysis of Expression Profiling of the FGF Genes 

In our previous studies, we have built numerous transcriptome databases of *S. schlegelii* for embryos at different developmental stages and different adult tissues [31]. Here, we utilized a total of 89 transcriptomes to demonstrate the expression profiles of *S. schlegelii* FGF genes based on the TPM (Transcripts Per Million) value. Previously, we established an in vitro muscle cell line from *S. schlegelii* with the capacity to differentiate into myotubes in vitro and built 16 transcriptomes of muscle cells during the in vitro differentiation process [65]. Then, the TPM value of FGF family genes was extracted. Moreover, the expression profile of FGF family genes during gonadal development was identified based on the transcriptome data, which was built in our previous study [66]. All heatmaps were drawn by TBtools [67].

### 4.4. qRT-PCR Validation of FGF Expression and Statistical Analysis

Total RNA was isolated using TRIzol reagent (SparkJade, Harbin, China) according to the standard protocol. Then, the genomic DNA removal and cDNA synthesis were performed using All-In-One 5X RT MasterMix (abm, Zhenjiang, China). Primers used for qRT-PCR were designed by Integrated DNA Technologies (http://sg.idtdna.com/pages/home (31 July 2022)) (Table S1). The *eif5a1* gene was used as the reference gene for standardizing the expressions of genes under testing [68]. The qRT-PCR analysis was performed under conditions as described previously [33]. The relative expressions of tested genes were calculated using the 2^−ΔΔCt^ comparative Ct method. Statistical analysis of qRT-PCR results was performed using SPSS20.0, and differences with *p* < 0.05 were considered to be significant. 

### 4.5. In Situ Hybridization

The ISH probes for *FGF1* were synthesized using a digoxigenin (DIG)-labeled RNA labeling kit (Roche, Berlin, Germany) based on the standard protocol. The samples of testis for ISH were fixed in 4% paraformaldehyde for 24 h. Then, the samples were dehydrated with serial methanol (30%, 50%, 70%, 90%, and 100%). After being cleared in xylene and embedded in paraffin, the samples were sectioned at 5 μm. ISH experiment was performed following the detailed protocols of our lab. The results were viewed and imaged by microscope (Nikon, Tokyo, Japan).

### 4.6. Vector Construction, Expression, and Purification of rSsFGF1 Protein

The vector construction, expression, and purification of FGF protein were carried out as described previously. In brief, the coding sequence of FGF1 was amplified with specific primers (Table S1) and then inserted into the pET-32a vector (Novagen, Madison, WI, USA). After validation by sequencing, the recombinant vector pet32a-FGF1 was transformed into E. coli BL21 and cultured in 37 ℃ for 12 h. Then, the culture was diluted with LB (1:100) and incubated at 37 °C until OD value = 0.6 and then treated with isopropyl-β-D-thiogalactoside (0.01 mM) for 20 h at 16 °C. After centrifuging at 8000 rpm at 4 °C for 15 min, the bacterial cells were harvested, re-suspended in PBS, and disintegrated by ultrasonication on ice. After centrifuging at 12,000 rpm at 4 °C for 20 min, the supernatant was loaded onto a Ni-NTA resin column (Sangon, Shanghai, China). The column was then washed with PBS containing serial imidazole (20 mmol/L, 30 mmol/L, 50 mmol/L, 200 mmol/L, and 500 mmol/L). After verification using SDS-PAGE, the protein diluted in PBS containing 200 mmol/L and 500 mmol/L imidazole was collected. After removing imidazole by Ultrafiltration Spin Columns (Beyotime, Shanghai, China), the protein concentration was examined with BCA Protein Assay Kit (CWBIO, Taizhou, China) following standard protocols.

### 4.7. Testis Culture In Vitro and FGF1 Protein Treatment

The testis culture in vitro was performed based on the system established in a previous study [69]. In brief, three healthy adult male *S. schlegelii* were anesthetized with MS222 (Sigma, Saint Louis, MO, USA), and testes were isolated. After being washed in PBS for 15 min, the testes were clipped into 10 mm^3^ blocks and then transferred into a 12-well culture plate and placed on a 0.25 cm^2^ nitrocellulose membrane, which rests on a 1.2 mL agarose cylinder placed in basal medium ((DMEM/F12 medium supplemented with penicillin–streptomycin, 10% fetal bovine serum, and non-essential amino acid). After incubation for 24 h in 28 ℃, the experiment group was added to FGF1 protein with different concentrations (10 ng/mL, 20 ng/mL and 50 ng/mL), and the control group was treated with an equal volume of PBS. After 48 h, the samples for RNA extraction and qRT-PCR were collected. Each experiment was performed in triplicate. 

## 5. Conclusions

In this study, we identified and characterized 24 FGF genes, and further expression analysis demonstrated that they were differentially involved in adult tissues and embryo developmental stages. Then, nine FGF genes were found to respond to myoblast differentiation in vitro and were involved in muscle development during the juvenile period and muscle injury repair. Furthermore, our results show that FGF genes were dynamically expressed during gonad development, and several FGF genes showed biased expressions in the testis or the ovary. Finally, *FGF1* was found to be extensively expressed in intestinal and sertoli cells in testis, and exogenous FGF1 protein was proven to function in germ-cell proliferation and differentiation. In sum, our work lays the foundation for further studies into muscle-growth regulation and gonad development of large teleost fishes and will be of benefit for the aquaculture industry.

## Figures and Tables

**Table 1:** The detailed information of FGF genes in *S. schlegelii*. Abbreviations for protein locations are: N, nucleus; C, cytoplasm; Es, extracellular space.

Gene	Chromosome	ORF	Amino Acid	Mw(kda)	PI	Location
FGF7	2	609	203	23.75	9.32	Es
FGF19	2	636	212	23.3	7.13	Es
FGF4	2	567	189	20.95	10.5	Es
FGF3	2	750	250	28.27	10.75	Es, N
FGF20a	3	636	212	23.56	7.16	Es
FGF18a	3	678	226	25.82	10.64	Es
FGF2	3	468	156	17.12	9.75	Es
FGF16	3	612	204	23.12	9	Es
FGF1a	3	474	158	17.8	6.2	Es
FGF13a	3	741	247	27.75	10.08	C, N
FGF13b	4	759	253	27.98	10.24	C, N
FGF1b	4	612	204	22.31	8.75	Es
FGF20b	5	636	212	23.56	7.16	Es
FGF10b	6	672	224	24.41	10.08	N
FGF12	7	669	223	25.09	9.26	C, N
FGF17	8	645	215	25.03	10.62	Es
FGF6a	9	648	216	23.96	9.54	Es
FGF8	15	417	139	16.36	10.56	Es
FGF23	16	801	267	29.93	5.67	Es
FGF6b	16	615	205	23.25	9.88	Es
FGF10a	17	741	247	27.27	10.38	N
FGF14	20	783	261	29.06	9.26	C, N
FGF11	23	753	251	28.29	10.01	C, N
FGF14-like	24	858	286	32.2	10.76	Es

## Data Availability

Not applicable.

## Supplementary Materials

The following supporting information can be downloaded at: https://www.mdpi.com/article/10.3390/ijms24043626/s1.
