# Supplementary material for: Identification, Characterization and Functional Analysis of Fibroblast Growth Factors in Black Rockfish (Sebastes schlegelii)
Source: Int J Mol Sci. 2023 Feb 11;24(4):3626. doi: 10.3390/ijms24043626 (PMC9958866; doi:10.3390/ijms24043626)
Supplement: Supplementary file 1 [file ijms-24-03626-s001.zip › ijms-2086513-supplementary.pdf]

Table S1. The primers used in this study.

Primers	Sequences(5'-3')	Tm(°C)	use
FGF10a qRT Fw	TCCTCCGCCTTCTTACTATC	60	qRT-PCR
FGF10a qRT Rv	ACACACACATACACACACTC	60	qRT-PCR
FGF19 qRT Fw	CAGTGGATCTAGGATGTGTTG	60	qRT-PCR
FGF19 qRT Rv	GTCTGTCGGAGACGTAGAT	60	qRT-PCR
FGF1b qRT Fw	ACACAGAGAGAGGGAGAAAG	60	qRT-PCR
FGF1b qRT Rv	TGCAACGACAGAGGAGAT	60	qRT-PCR
FGF2 qRT Fw	CTGGCTTCTGACTGTGTAAG	60	qRT-PCR
FGF2 qRT Rv	GGGTTTCGTGTGCTTAGTT	60	qRT-PCR
FGF6a qRT Fw	CTCTCTGCCCTTGTCTTATTG	60	qRT-PCR
FGF6a qRT Rv	CTCTCTCACACACACTCTCT	60	qRT-PCR
FGF14 qRT Fw	CCGCCTTCTCTGGTAAATAG	60	qRT-PCR
FGF14 qRT Rv	GTAGACCATCTGGGTCAAAG	60	qRT-PCR
FGF1a qRT Fw	GCACTGGGATGGATTGTT	60	qRT-PCR
FGF1a qRT Rv	TTCGGTCTTGACCCATTTG	60	qRT-PCR
FGF18a qRT Fw	CCCTTCATAGGCCAGAAATAC	60	qRT-PCR
FGF18a qRT Rv	GGTCCAGTTGTAATCCCATAC	60	qRT-PCR
FGF7 qRT Fw	ACCAGGAGGGAGCATATAG	60	qRT-PCR

FGF7 qRT Rv	GGAGCGTGATGGTAATGAG	60	qRT-PCR
nanos2-qRT-Fw	GGACTAGTGATGGGAATTTAGG	60	qRT-PCR
nanos2-qRT-Rv	CTAACAAGCAGACACCTGTC	60	qRT-PCR
piwi1-qRT-Fw	GACCTCCTCTTCTACCTATGAG	60	qRT-PCR
piwi1-qRT-Rv	GGGCTATCTCACACTACAAAC	60	qRT-PCR
vasa-qRT-Fw	CGACTGCAGATGAAGAAGAA	60	qRT-PCR
vasa-qRT-Rv	GCGTCATAAACGAGACCTG	60	qRT-PCR
sycp3-qRT-Fw	AGGGTAGTGGTTAGAAGGTAG	60	qRT-PCR
sycp3-qRT-Rv	GTTCAAGGTCGTGTCTAGAAG	60	qRT-PCR
mns1-qRT-Fw	GATGGCTGGGAAGAAGATG	60	qRT-PCR
mns1-qRT-Rv	CAGCAGCACAGTCCTAATAA	60	qRT-PCR
spo11-qRT-Fw	GGTATGAGCCCAATGCTGCTG	60	qRT-PCR
spo11-qRT-Rv	ATTACAGGGCTTTAATGATAG	60	qRT-PCR
pcna-qRT-Fw	TTCAGTGCTCTGACTCTTTG	60	qRT-PCR
pcna-qRT-Rv	GCGAAACCACAGGAAGTAT	60	qRT-PCR
mki67-qRT-Fw	GTCGTTGATGACCACTTAAAGG	60	qRT-PCR
mki67-qRT-Rv	CTACTGTGTCCACTTTAGGCTGT	60	qRT-PCR
top2a-qRT-Fw	ACTCTCCAAGTCAGTTTTAG	60	qRT-PCR
top2a-qRT-Rv	CTTAGTAACACACAAGTAT	60	qRT-PCR

eif5a1-qRT-Fw	CTTTGCTCTGGTTCCTGAGTGG	60	qRT-PCR
eif5a1-qRT-Rv	AGCTTTGACATGCTGGGGTG	60	qRT-PCR
FGF1 ISH Fw	**ATTTAGGTGACACTATAGAAGAGGCTGCTGGAGATCTGTTTGA**	58	ISH
FGF1 ISH Rv	**TAATACGACTCACTATAGGGAGATCTCTCTGTGTCTCCCTTTCT**	58	ISH

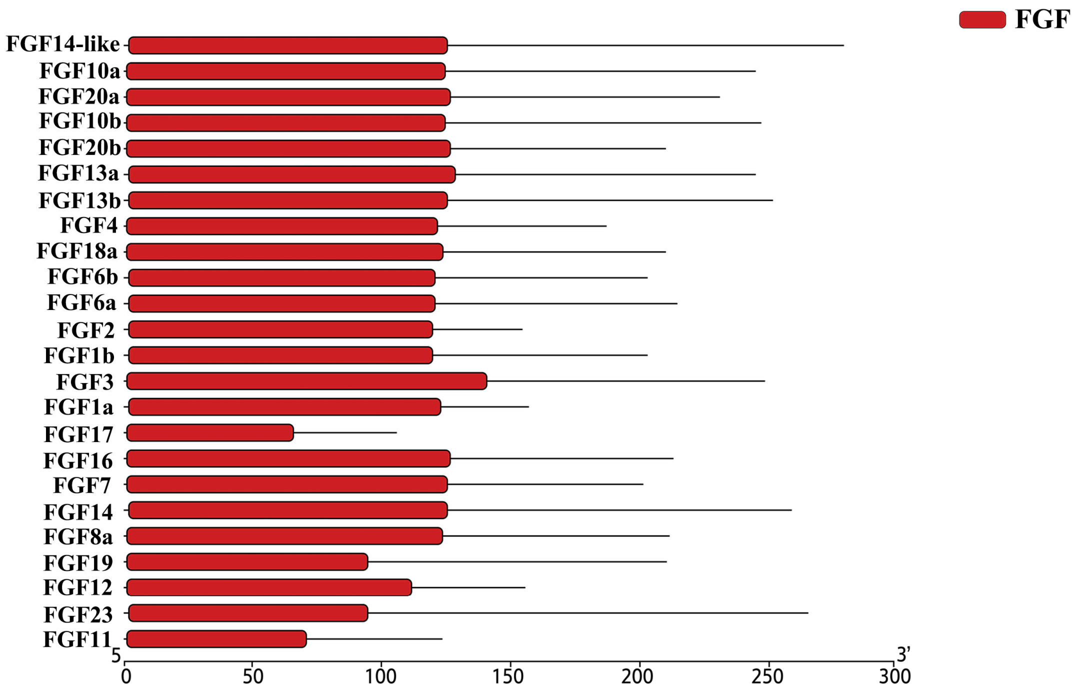

Figure S1 Domain analysis of FGF genes in S.schlegelii. The FGF domain was shown in red. The length of black line represented the number of amino acids of each protein in scale.

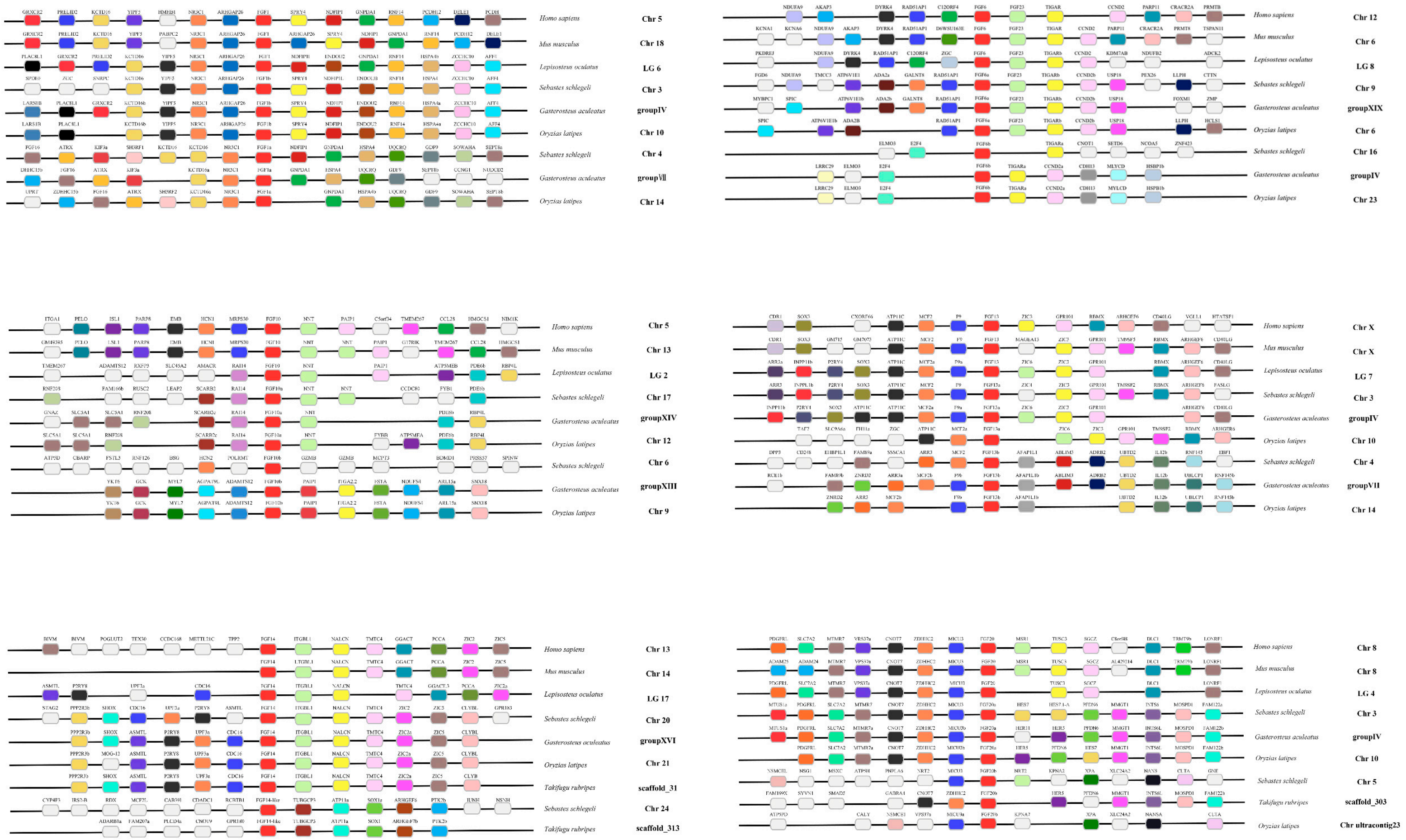

Figure S2 Syntenic analysis of FGF genes on the chromosome or scaffold. The pentagons in different colors represented different genes.

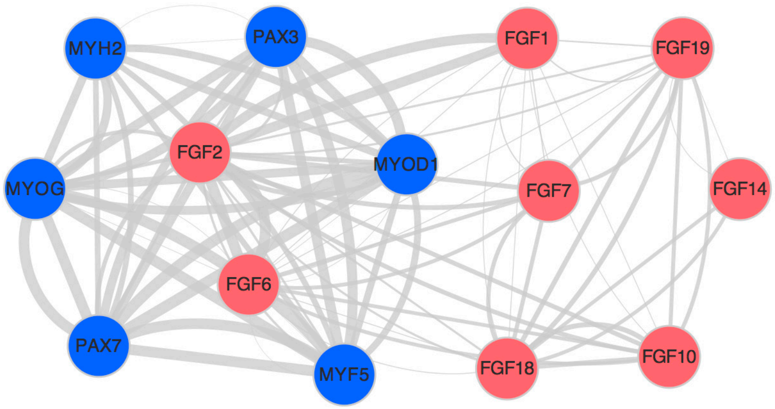

Figure S3 The protein-protein interaction between FGF genes and muscle cell related genes. The width of lines represented the strength of interaction.

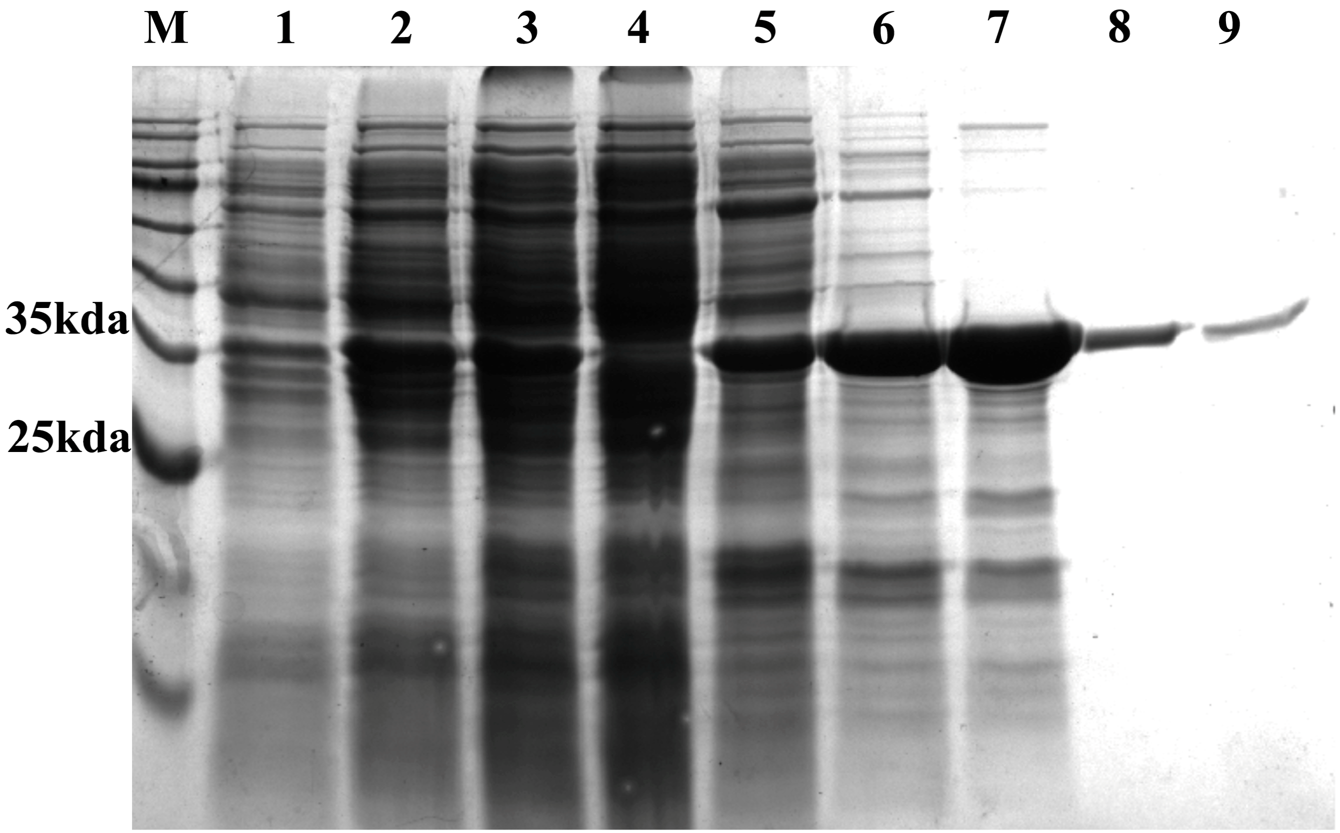

Figure S4 Prokaryotic expression and purification of S.schlegelii FGF1 protein. M: marker; lane 1: negative control (without induction); lane 2: total cellular extracts from 1ml IPTG-induced E. coli BL21 (DE3)-containing expression vector; lane 3: supernatant fluid of cellular extracts from 500 ml IPTG-induced E. coli BL21 (DE3)-containing expression vector; lane 4: the supernatant fluid after flowing through Ni-NTA resin column; lane 5 - lane 9: the protein washed down from Ni-NTA resin column with 20mm imidazole, 30mm imidazole, 50mm imidazole, 200mm imidazole and 500mm imidazole.
